# Diagnostic yield and variant spectrum of whole-exome sequencing in Iranian probands with congenital and early-onset ocular disorders

**DOI:** 10.1016/j.ymgmr.2026.101339

**Published:** 2026-07-20

**Authors:** Ali Asadi, Seyed Ataollah Sadat Shandiz, Amirhossein Ebrahimi, Zahra Sadat Hosseini, Niyousha Shirsalimi, Hossein Neamatzadeh, Ahmad Ebrahimi

**Affiliations:** a Department of Biology, CT.C., Islamic Azad University, Tehran, Iran; b Department of Computer Engineering, Shiraz University, Shiraz, Iran; c Faculty of Medicine, Hamadan University of Medical Sciences, Hamadan, Iran; d Department of Art, Faculty of Shariati, National University of Skills, Tehran, Iran; e Hematology and Oncology Research Center, Non-Communicable Diseases Research Institute, Shahid Sadoughi University of Medical Science, Yazd, Iran; f Genia Medical Genetics Center, Tehran, Iran

**Keywords:** Inherited ocular disorders, Whole-exome sequencing, Inherited retinal disease, Consanguinity, Genetic counselling, Precision medicine

## Abstract

**Background:**

Inherited ocular disorders are a leading cause of early-onset visual impairment, particularly in populations with high consanguinity such as Iran, where a substantial proportion of affected individuals remain without a molecular diagnosis after conventional evaluation. We aimed to determine the diagnostic yield and variant spectrum of whole-exome sequencing (WES) in Iranian probands with congenital or early-onset ocular disorders that were genetically unresolved by prior testing.

**Methods:**

Thirty unrelated probands were recruited consecutively (July–September 2024). Genomic DNA underwent exome capture (Agilent SureSelect V7) and paired-end sequencing (Illumina NovaSeq 6000). Reads were cleaned with SOAPnuke, aligned with BWA-MEM, and variants were called with GATK HaplotypeCaller and DeepVariant, filtered by GATK VQSR, and annotated against public and Iranian-specific databases (GEMIRAN, IRANOM). Variants were classified per ACMG/AMP criteria. Diagnostic yield was defined as the proportion of probands with a causative or candidate variant concordant with phenotype.

**Results:**

A causative or strong candidate variant concordant with the phenotype was identified in 21 of 30 probands (diagnostic yield 70.0%; 95% CI 50.6–85.3%). Pathogenic or likely-pathogenic variants were found in established genes including *ABCA4, USH2A, RP1, CRB1, CEP290, GUCY2D, CYP1B1* and *TYR*. Among the identified genotypes, 54% were homozygous and 12% hemizygous (X-linked), consistent with consanguinity in 11/30 (36.7%) families; 35% were single heterozygous findings in autosomal-recessive genes, interpreted as incomplete genotypes pending detection of a second allele. Onset was infantile in 73% of probands.

**Conclusions:**

WES is an effective first-tier test for congenital and early-onset ocular disorders in the Iranian population, resolving roughly 70% of previously undiagnosed probands. Single-allele findings in recessive genes indicate that complementary copy-number and structural-variant analysis, deep-intronic assessment, periodic reanalysis, and reflex whole-genome sequencing are needed to maximise yield and support accurate genetic counselling.

## Highlights

• Whole-exome sequencing for 30 probands with congenital or early-onset ocular disorders unresolved by conventional testing.
• Pathogenic variants in *ABCA4*, *USH2A*, *RP1*, *CRB1*, *CEP290*, *CYP1B1*, *TYR*; mostly homozygous, indicating recessive inheritance.
• Confirm the clinical utility of whole-exome sequencing in genetically heterogeneous and consanguineous populations.
• Several heterozygous variants in recessive genes suggested partially detected biallelic genotypes.

## 1 Introduction

Inherited eye diseases comprise a broad spectrum of monogenic conditions and remain a leading cause of visual impairment worldwide [1]. Disorders such as retinitis pigmentosa (RP), Stargardt disease, and Leber congenital amaurosis (LCA) manifest from birth to early adulthood, with phenotypes ranging from night blindness to profound vision loss [2], [3]. Advances in next-generation sequencing (NGS) have clarified inheritance patterns, identified disease loci, and enabled precision diagnostics for these conditions [4], [5].

Whole-exome sequencing (WES) has become a key diagnostic test for inherited ocular disorders, outperforming targeted panels by capturing rare, private, and compound-heterozygous variants [6], [7]. In populations with high consanguinity such as Iran, autosomal-recessive disorders are over-represented and founder alleles require population-specific interpretation; Iranian studies report diagnostic yields exceeding 70% in consanguineous families with inherited retinal diseases (IRDs), with recurrent pathogenic variants in genes including *ABCA4, RHO, RPGR, EYS* and *USH2A* [8], [9], [10]. These observations underscore the value of WES for early detection and counselling while emphasising the need for local allele-frequency resources to enhance interpretive precision [11].

Iranian and comparative studies consistently show high diagnostic yield, strong genotype–phenotype correlations, and population-specific variant patterns. Ardehaie et al. reported prevalent pathogenic and novel IRD variants and a higher frequency of syndromic IRDs in eastern Iran [12]; Zamani et al. reported approximately 82% yield from WES in southwest Iran [13]; and an integrated WES and autozygosity-mapping study of 192 Iranian families achieved a 72.9% molecular diagnosis [14]. Chitsazian et al. linked *CYP1B1* mutations to earlier, more severe primary congenital glaucoma in Iranian patients [15], and comparable international series report broad allelic heterogeneity across IRD genes [16], [17].

Despite this progress, the genetic architecture of congenital and early-onset ocular disorders in Iranian families remains incompletely characterised, and many patients are still undiagnosed after conventional evaluation. The primary aim of this study was to determine the diagnostic yield and variant spectrum of WES in a consecutive series of 30 Iranian probands whose congenital or early-onset ocular disorders were unresolved by prior ophthalmic and genetic testing. Secondary aims were to characterise the inheritance patterns and contribution of consanguinity, to quantify single-allele (incomplete) genotypes, and to evaluate incidental and secondary findings. Beyond confirming the utility of WES, this cohort adds value by applying Iranian-specific allele-frequency resources (GEMIRAN and IRANOM) and by transparently quantifying the proportion of partially resolved genotypes that would benefit from reflex genomic testing — a question of direct relevance to genetic-counselling practice in Iran and the wider Middle East.

## 2 Materials and methods

### 2.1 Study design and participants

This was a single-centre, prospective case series. Thirty unrelated probands from Iranian families with congenital or early-onset ocular disorders were recruited consecutively from the Eye Research Center of Shahid Beheshti University of Medical Sciences and Dr. Ebrahimi Medical Genetics Center between 9 July and 15 September 2024. Inclusion criteria were: (i) a congenital or early-onset ocular phenotype; (ii) prior clinical ophthalmic evaluation (fundus examination, optical coherence tomography, and electroretinography as appropriate) and, where available, prior targeted testing that had not established a molecular diagnosis; and (iii) informed consent. Exclusion criteria were an established prior molecular diagnosis and acquired or clearly non-genetic causes of visual impairment. “Early-onset” was defined as symptom onset within the first two decades of life, and “genetically unresolved” as the absence of a confirmed molecular diagnosis after this evaluation.

### 2.2 Operational definitions

A case was classified as solved when pathogenic/likely-pathogenic variant(s) were identified with a zygosity consistent with the inheritance mode of a gene matching the phenotype; partially solved when a single heterozygous pathogenic/likely-pathogenic variant was found in an autosomal-recessive gene concordant with the phenotype; and unsolved when no concordant causative variant was identified. Diagnostic yield was defined as (solved + partially solved)/total probands. Incidental and secondary findings were defined per ACMG SF v3.2 [18] and distinguished from incomplete recessive genotypes, which involve a single allele in a gene matching the primary phenotype.

### 2.3 Sample collection and sequencing

Peripheral-blood genomic DNA was extracted and quantified using a DeNovix spectrophotometer; approximately 300 ng per sample was submitted for library preparation. Exome libraries were prepared with the Agilent SureSelect Human All Exon V7 kit and sequenced on an Illumina NovaSeq 6000 platform (2 × 150 bp), targeting a mean on-target depth of ≈99–100× with ≈95% of target bases covered at ≥20× (per-sample metrics, Supplementary Table S1).

### 2.4 Bioinformatic pipeline and variant calling

Raw reads were quality-controlled with SOAPnuke (BGI) to remove adaptors, contaminants, and low-quality reads prior to alignment, then aligned to GRCh38/hg38 with BWA-MEM (v0.7.17). After duplicate marking and base-quality-score recalibration (GATK v4.2), variants were called with GATK HaplotypeCaller and DeepVariant (v1.4) and filtered by GATK Variant Quality Score Recalibration (VQSR).

### 2.5 Annotation, filtering and ACMG interpretation

Variants were annotated with population databases (gnomAD v4) and Iranian-specific reference genomes (GEMIRAN, IRANOM), and prioritised by retaining rare (minor-allele frequency ≤ 0.01), exonic or splice-region variants predicted deleterious. In-silico predictors (CADD, REVEL, SpliceAI, AlphaMissense) supported pathogenicity assessment. Variants were classified following the ACMG/AMP framework [19], applying PVS1 for null variants in genes with an established loss-of-function mechanism together with PS/PM/PP and BS/BP criteria; evidence was drawn from population frequency, computational prediction, and, where available, segregation. Each variant was independently reviewed by two analysts, with discordant calls resolved by consensus. Candidate variants were assessed for segregation in families where parental samples were available.

### 2.6 Copy-number analysis and statistics

Dedicated copy-number and structural-variant calling was not performed; related statements are framed as limitations and future directions. Categorical variables are summarised as counts and percentages, and diagnostic yield is reported with an exact (Clopper–Pearson) 95% confidence interval. Consistent with a descriptive consecutive case series, no a-priori sample-size calculation or formal hypothesis testing was undertaken.

### 2.7 Ethics and consent

The protocol was approved by the institutional Ethics Committee (IR.IAU.CTB.REC.1403.097) and conducted in accordance with the Declaration of Helsinki. Written informed consent was obtained from all participants or, for those under 18 years, from a parent or legal guardian. Consent explicitly covered the return of secondary findings, which were reported in line with ACMG SF v3.2 [18]. Data were anonymised to protect privacy.

## 3 Results

### 3.1 Cohort demographics

Thirty unrelated probands were studied (Table 1). Onset was infantile in 22/30 (73.3%), within 3–10 years in 6/30 (20.0%), and in the second decade in 2/30 (6.7%). Parental consanguinity was documented in 11/30 (36.7%) families. The cohort spanned non-syndromic IRDs (RP, LCA, cone–rod dystrophy) and syndromic entities (Bardet–Biedl, Aarskog–Scott, oral–facial–digital, and Wiedemann–Steiner syndromes), reflecting referral of clinically heterogeneous, previously unresolved cases.

**Table 1:** Clinical and demographic characteristics of the 30 probands.

Proband ID	Consanguinity	Age of onset	Primary clinical diagnosis	Confirmed phenotype	Causative variant	Incidental finding
EX-28ZA	Yes	Infantile	Impaired vision, AR	No definitive diagnosis	No	Yes
EX-9-23MM	No	Infantile	Strabismus, AR	No definitive diagnosis	No	Yes
EX-4NF	No	Infantile	Aniridia, AD	Aniridia, ONH, AD	Yes	Yes
06-45 J	No	5–10 y	RP/BBS, AR	BBS, AR	Yes	Yes
EX-33 dB	No	3–5 y	Impaired vision, XLR	XLPDR, XL	Yes	Yes
04-05S	No	Infantile	OFD, XLD	OFD, XLD	Yes	No
EX-30RN	No	Infantile	Syndromic eye disorder, AR	No definitive diagnosis	No	Yes
EX-22R	No	Infantile	Impaired vision, AR	No definitive diagnosis	No	Yes
EX-41HA	No	Infantile	RP/BBS, AR	Bardet–Biedl syndrome, AR	Yes	Yes
EX-12RM	No	Infantile	Syndromic eye disorder, AR	No definitive diagnosis	No	Yes
04-22ZAG	Yes	Infantile	MD/CSNB, AR	RP, AR	Yes	No
06-54BO	No	5–10 y	Retinal atrophy, AR	CRD 3B, AR	Yes	Yes
04-21ZOG	Yes	Infantile	MD/CSNB, AR	RP, AR	Yes	No
EX-24AF	No	2nd decade	RP, AD	RP, AD	Yes	No
EX-9ZB	No	Infantile	Syndromic impaired vision, AD	WDSTS, AD	Yes	Yes
1209KM	Yes	Infantile	Impaired vision, AR	LCA, AR	Yes	No
EX-02-09	Yes	Infantile	Myopia with microcephaly, AR	No definitive diagnosis	No	Yes
08-41ZP	No	Infantile	RP/cataract, AR	RP, CTRCT9, AR	Yes	Yes
EX-48TP	No	Infantile	Impaired vision, AR	No definitive diagnosis	No	Yes
EX-13FR	Yes	Infantile	Impaired vision, AR	LCA, AR	Yes	No
EX-16AZ	No	Infantile	Impaired vision, XLR	AAS, XL	Yes	No
EX-52BM	Yes	3–5 y	AS, AD	Alport syndrome, AD	Yes	No
11-21SR	Yes	Infantile	Impaired vision, AR	No definitive diagnosis	No	Yes
EX-41HJ	No	Infantile	Impaired vision, AR	No definitive diagnosis	No	Yes
EX-12-61ZA	No	Infantile	Albinism/nystagmus, AR	OCA, AR	Yes	No
EX-37 MJ	No	Infantile	Nystagmus, AR	CMYO6, AR	Yes	No
EX-12AS	Yes	Infantile	Pigmentation abnormality, AR	Pigmentation abnormality, AR	Yes	Yes
06-43HP	Yes	5–10 y	RP/BBS, AR	BBS, AR	Yes	No
07-24MF	Yes	3–5 y	RP/BBS, AR	BBS, AR	Yes	No
EX-14MKH	No	2nd decade	Usher syndrome, AR	Usher syndrome, digenic	Yes	No

Abbreviations: AR, autosomal recessive; AD, autosomal dominant; XL, X-linked; XLR, X-linked recessive; XLD, X-linked dominant; RP, retinitis pigmentosa; ONH, optic nerve hypoplasia; BBS, Bardet–Biedl syndrome; XLPDR, X-linked reticulate pigmentary disorder; OFD, oral–facial–digital syndrome; CRD, cone–rod dystrophy; WDSTS, Wiedemann–Steiner syndrome; LCA, Leber congenital amaurosis; CTRCT9, early-onset cataract; AAS, Aarskog–Scott syndrome; OCA, oculocutaneous albinism; CMYO6, congenital myopathy type 6; MD, macular degeneration; CSNB, congenital stationary night blindness; US, Usher syndrome.

### 3.2 Diagnostic yield

A causative or strong candidate variant concordant with the phenotype was identified in 21/30 probands, a diagnostic yield of 70.0% (95% CI 50.6–85.3%). Most represented definitive (biallelic or appropriately hemizygous) diagnoses, while a subset were partially solved on the basis of a single heterozygous pathogenic/likely-pathogenic allele in an autosomal-recessive gene. Nine probands (30.0%) remained unsolved.

### 3.3 Variant spectrum and inheritance

Pathogenic, likely-pathogenic, and uncertain variants were distributed across a broad range of IRD genes (Table 2), comprising loss-of-function (stop-gain, frameshift), splice-site, and deleterious missense alterations. Among the 26 gene-level genotypes in Table 2, 14 (54%) were homozygous and 3 (12%) hemizygous X-linked, so that two-thirds were consistent with the homozygosity expected under consanguinity; the remaining 9 (35%) were single heterozygous findings in recessive genes.

**Table 2:** Genotype–phenotype correlations.

ID	Gene / HGVS	Associated phenotype	Mutation type	Zygosity	ACMG	ClinVar	PMID
1	RP1 chr8 NM_006269.2:c.788-1G > A	Retinitis pigmentosa, AR	Splice site	Hom	LP	LP	33576794
2	PAX6 chr11 NM_001368929.2:c.730_731del p.Lys244Glyfs*7	Aniridia, ONH, AD	Frameshift del	Het	LP	Conflicting	38248310
3	BBS10 chr12 NM_024685.4:c.947del p.Pro316Glnfs*3	Bardet–Biedl syndrome, AR	Frameshift del	Hom	LP	–	29666954
4	POLA1 chrX NM_016937.4:c.3550 T > G p.Ser1184Ala	XLPDR, XL^a^	Missense	Hem	VUS	VUS	38165470
5	OFD1 chrX NM_001330209.2:c.710del p.Lys237Serfs*6	OFD syndrome, XL^b^	Frameshift del	Hem	P	P	30401917
6	CFAP418 chr8 NM_177965.4:c.394 T > C p.Cys132Arg	Bardet–Biedl syndrome, AR	Missense	Hom	VUS	VUS	27008867
7	KCNV2 chr9 NM_133497.4:c.995_996dup p.Ser333Alafs*122	CRD 3B, AR	Frameshift dup	Hom	P	P	29210963
8	ATAD3A chr1 NM_018188.5:c.178 A > T p.Lys60X	Harel–Yoon syndrome (OMIM 617183), AD/AR^c^	Stop-gain	Het	LP	–	36856321
9	ABCA4 chr1 NM_000350.3:c.1 A > G p.Met1?	RP19, AR^d^	Start-loss	Het	VUS (single allele)	P*	24265693
10	KMT2A chr11 NM_005933.4:c.5993_5996del p.Phe1998Trpfs*8	WDSTS, AD^e^	Frameshift del	Het	P	P	34828665
11	GUCY2D chr17 NM_000180.4:c.2828dup p.Arg944Alafs*27	LCA, AR	Frameshift dup	Hom	LP	–	32821499
12	GOLGA8M chr15 NM_001282468.2:c.1321C > T p.Gln441X	VUS — not an established retinal-disease gene^f^	Stop-gain	Hom	VUS	–	17573966
13	CRYAA chr21 NM_000394.4:c.124 T > C p.Ser42Pro	CTRCT9, AR	Missense	Hom	VUS	–	30340470
14	CRB1 chr1 NM_001193640.2:c.3158G > A p.Cys1053Tyr	RP12, AR	Missense	Hom	LP	–	29391521
15	ADGRV1 chr5 NM_032119.4:c.14197C > T p.Gln4733X	Usher syndrome type 2C, AR^g^	Stop-gain	Het	LP	–	37422204
16	PDZD7 chr10 NM_001195263.2:c.1079G > A p.Gly360Asp	Usher syndrome type 2C (digenic)^g^	Missense	Het	VUS	VUS	20440071
17	BBS12 chr4 NM_001178007.2:c.265_266del p.Leu89Valfs*11	Bardet–Biedl syndrome 12, AR	Frameshift del	Hom	P	P	31888296
18	CEP290 chr12 NM_025114.4:c.6870del p.Gln2291Lysfs*10	LCA, AR	Frameshift del	Hom	LP	–	33595255
19	FGD1 chrX NM_004463.3:c.527dup p.Leu177Thrfs*40	AAS (Aarskog–Scott), XL^h^	Frameshift dup	Hem	P	P	35388608
20	SACS chr13 NM_014363.6:c.4343del p.Asn1448Thrfs*15	ARSACS, AR^i^	Frameshift del	Hom	LP	–	36458808
21	COL4A3 chr2 NM_000091.5:c.4882 T > G p.Ser1628Ala	Alport syndrome, AD^j^	Missense	Het	VUS	Conflicting	24052634
22	TUBA3D chr2 NM_080386.4:c.466C > T p.Arg156Trp	Keratoconus, AD^k^	Missense	Het	VUS	–	29051577
23	MKKS chr20 NM_170784.3:c.1622G > A p.Cys541Tyr	Bardet–Biedl syndrome 6, AR	Missense	Hom	VUS	–	10973238
24	TYR chr11 NM_000372.5:c.139G > T p.Gly47Cys	OCA1, AR^l^	Missense	Het	LP	–	32411182
	TYR chr11 NM_000372.5:c.1430G > A p.Trp477X	(compound heterozygous with above)	Nonsense	Het	LP	–	30472657
25	MYH2 chr17 NM_017534.6:c.5045G > A p.Arg1682His	CMYO6, AR^m^	Missense	Hom	VUS	VUS	36774715
26	MC1R chr16 NM_002386:c.623_626dup p.Met210Profs*30	Pigmentation variation (incidental)^n^	Frameshift dup	Hom	VUS	–	29316344

Abbreviations: Hom, homozygous; Het, heterozygous; Hem, hemizygous; P, pathogenic; LP, likely pathogenic; VUS, variant of uncertain significance. “—” indicates no ClinVar entry. * Single-allele finding (footnote d). PMIDs correspond to the cited variant/gene reports.

a POLA1: X-linked reticulate pigmentary disorder is a multisystem condition (skin, immune, corneal) with predominantly extra-ocular features; reported as hemizygous in this male proband.

b OFD1: oral–facial–digital syndrome; reported as hemizygous. Male survival may reflect hypomorphic alleles or mosaicism.

c ATAD3A: Harel–Yoon syndrome (OMIM 617183); inheritance may be autosomal dominant (frequently de novo) or, rarely, autosomal recessive; optic atrophy is a secondary feature.

d ABCA4: RP19 (OMIM 601718) is autosomal recessive and requires biallelic variants; the single start-loss allele detected here is non-diagnostic pending identification of a second allele.

e KMT2 A: Wiedemann–Steiner syndrome is a neurodevelopmental disorder; ocular features are secondary.

f GOLGA8M: not an established retinal-disease gene (OMIM/RetNet); reported as a variant of uncertain significance. The RP11 locus corresponds to PRPF31 on chromosome 19.

g ADGRV1/PDZD7: Usher syndrome type 2C; digenic inheritance requires variants in trans in both genes; phase confirmation is pending.

h FGD1: Aarskog–Scott syndrome is a developmental disorder with secondary ocular features; reported as hemizygous.

i SACS: autosomal recessive spastic ataxia of Charlevoix–Saguenay is a primary ataxia; retinal changes are secondary.

j COL4A3: Alport syndrome is a primary renal disorder; ocular features (e.g., lenticonus) are secondary.

k TUBA3D: keratoconus association rests on limited evidence and is classified as a variant of uncertain significance pending replication; this is a corneal, not retinal, phenotype.

l TYR: two heterozygous likely-pathogenic alleles were detected (compound heterozygous), potentially diagnostic for OCA1 if confirmed in trans.

m MYH2: congenital myopathy type 6 is a primary skeletal-muscle disorder with secondary ophthalmoplegia.

n MC1R: a pigmentation-variation gene, not a retinal-disease gene; reported as an incidental finding.

### 3.4 Genotype–phenotype correlations and illustrative cases

**Several probands showed tight genotype–phenotype concordance. Proband 06-45J** (syndromic RP) carried a homozygous frameshift in *BBS10* (c.947del), consistent with Bardet–Biedl syndrome and the comparatively high frequency of BBS in Middle Eastern cohorts [20], [21]. **Proband 1209KM** (infantile impaired vision, consanguineous) carried a homozygous frameshift in *GUCY2D***, concordant with LCA. Proband EX-33DB** (X-linked pattern) carried a hemizygous *POLA1* variant; because X-linked reticulate pigmentary disorder is a multisystem condition with secondary ocular features, this finding was interpreted with caution. These examples show how concordant genotypes directly informed counselling and, where relevant, surveillance for extra-ocular features.

### 3.5 Single-allele and incidental findings

Single heterozygous pathogenic/likely-pathogenic variants in autosomal-recessive IRD genes (e.g., *USH2A, ABCA4, RP1, CNGA3, PDE6A, MERTK*) were detected in several probands (Table 3); most likely represent incompletely detected biallelic genotypes rather than true secondary findings, and were flagged for reflex copy-number and segregation analysis. A minority of variants lay in genes unrelated to the ocular phenotype (e.g., *MC1R*) and were handled as incidental findings according to phenotype correlation, consent, and ACMG recommendations [18]. Table 4 consolidates the full set of clinically relevant variants identified across the cohort.

**Table 3:** Single-allele and incidental variants identified by exome sequencing.

Gene / HGVS	Phenotype	Variant type	Zygosity	ACMG	ClinVar	AlphaMissense
TRPM1 (AR) chr15 NM_001252020.2:c.1140 + 1G > A	CSNB	Splice donor	Het	P	LP	SAS
TRPM1 chr15 NM_001252020.2:c.617_642del p.Gly206Glufs*2		Frameshift del		LP	–	–
CACNA2D4 (AR) chr12 NM_172364.5:c.1940 + 1G > A	CSNB, RCD4	Splice donor	Het	LP	VUS	SAS
CACNA2D4 chr12 NM_172364.5:c.2552-1G > A		Splice acceptor		P	VUS	SAS
PDE6A (AR) chr5 NM_000440.3:c.603_607del p.Phe203Glnfs*4	RP43	Frameshift del	Het	LP	–	–
CYP1B1 (AR) chr2 NM_000104.4:c.182G > A p.Gly61Glu	Congenital glaucoma	Missense	Het	LP	P	D
CYP1B1 chr2 NM_000104.4:c.1330C > T p.Arg444X		Stop-gain		P	P	–
TYR (AR) chr11 NM_000372.5:c.1352 A > G p.Tyr451Cys	OCA type IA/IB	Missense	Het	P	P	B
CNGA3 (AR) chr2 NM_001298.3:c.827 A > G p.Asn276Ser	Achromatopsia 2	Missense	Het	P	LP	B
MERTK (AR) chr2 NM_006343.3:c.2302G > A p.Ala768Thr	RP38	Missense	Het	P	LP	D
ABCA4 (AR) chr1 NM_000350.3:c.3898C > T p.Arg1300X	CRD/RP	Stop-gain	Het	P	P	–
USH2A (AR) chr1 NM_206933.4:c.6937G > T p.Gly2313Cys	RP39 / Usher 2A	Missense	Het	P	P	U
SLC24A1 (AR) chr15 NM_001254740.2:c.847G > A p.Val283Met	CSNB (complete)	Missense	Het	VUS	–	D
RP1 (AR) chr8 NM_006269.2:c.5797C > T p.Arg1933X	RP1	Stop-gain	Het	P	P	–
IQCB1 (AR) chr3 NM_001319107.2:c.766 + 1G > A	LCA / Senior–Løken	Splice donor	Het	LP	–	SAS
CFI (AR) chr4 NM_001331035.2:c.1305del p.Asp436Ilefs*49	AMD risk	Frameshift del	Het	LP	–	–
USP45 (AR) chr6 NM_001346024.3:c.1921_1925del p.Val641Glnfs*20	LCA19	Frameshift del	Het	LP	–	–
PCK2 (AR) chr14 NM_004563.4:c.133C > T p.Arg45X	PACG	Stop-gain	Het	LP	–	–

Abbreviations: AR, autosomal recessive; Het, heterozygous; P, pathogenic; LP, likely pathogenic; VUS, variant of uncertain significance; B, benign; D, deleterious; U, uncertain; SAS, splice-altering (strong); CSNB, congenital stationary night blindness; RCD, retinal cone dystrophy; RP, retinitis pigmentosa; OCA, oculocutaneous albinism; CRD, cone–rod dystrophy; LCA, Leber congenital amaurosis; AMD, age-related macular degeneration; PACG, primary angle-closure glaucoma. AlphaMissense and SpliceAI provide in-silico predictions.

**Table 4:** Full list of clinically relevant variants identified across the cohort.

Gene	Chr	cDNA	Protein	Function	ACMG	ClinVar	AlphaMissense	SpliceAI
*RP1*	8	c.788-1G > A	–	Splice acceptor	LP	LP	–	B
*RP1*	8	c.5797C > T	p.Arg1933X	Stop-gain	P	P	–	–
*TYR*	11	c.139G > T	p.Gly47Cys	Missense	LP	–	D	B
*TYR*	11	c.1430G > A	p.Trp477X	Nonsense	LP	–	–	B
*TYR*	11	c.1352 A > G	p.Tyr451Cys	Missense	P	P	B	B
*TRPM1*	15	c.1140 + 1G > A	–	Splice donor	P	LP	–	D
*TRPM1*	15	c.617_642del	p.Gly206Glufs*2	Frameshift del	LP	–	–	–
*CACNA2D4*	12	c.1940 + 1G > A	–	Splice donor	LP	VUS	–	D
*CACNA2D4*	12	c.2552-1G > A	–	Splice acceptor	P	VUS	–	D
*PDE6A*	5	c.603_607del	p.Phe203Glnfs*4	Frameshift del	LP	–	–	–
*CYP1B1*	2	c.182G > A	p.Gly61Glu	Missense	LP	P	D	B
*CYP1B1*	2	c.1330C > T	p.Arg444X	Stop-gain	P	P	–	B
*PAX6*	11	c.730_731del	p.Lys244Glyfs*7	Frameshift del	LP	Conflicting	–	B
*BBS10*	12	c.947del	p.Pro316Glnfs*3	Frameshift del	LP	–	–	B
*POLA1*	X	c.3550 T > G	p.Ser1184Ala	Missense	VUS	VUS	B	B
*OFD1*	X	c.710del	p.Lys237Serfs*6	Frameshift del	P	P	–	D
*CFAP418*	8	c.394 T > C	p.Cys132Arg	Missense	VUS	VUS	D	B
*KCNV2*	9	c.995_996dup	p.Ser333Alafs*122	Frameshift dup	P	P	–	–
*ATAD3A*	1	c.178 A > T	p.Lys60X	Stop-gain	LP	–	–	B
*ABCA4*	1	c.1 A > G	p.Met1?	Start-loss	VUS*	P	–	B
*ABCA4*	1	c.3898C > T	p.Arg1300X	Stop-gain	P	P	–	B
*KMT2A*	11	c.5993_5996del	p.Phe1998Trpfs*8	Frameshift del	P	P	–	B
*GUCY2D*	17	c.2828dup	p.Arg944Alafs*27	Frameshift dup	LP	–	–	B
*GOLGA8M*	15	c.1321C > T	p.Gln441X	Stop-gain	VUS	–	–	B
*ADGRV1*	5	c.14197C > T	p.Gln4733X	Stop-gain	LP	–	–	B
*PDZD7*	10	c.1079G > A	p.Gly360Asp	Missense	VUS	VUS	–	B
*CRYAA*	21	c.124 T > C	p.Ser42Pro	Missense	VUS	–	U	B
*CRB1*	1	c.3158G > A	p.Cys1053Tyr	Missense	LP	–	D	B
*BBS12*	4	c.265_266del	p.Leu89Valfs*11	Frameshift del	P	P	–	B
*CEP290*	12	c.6870del	p.Gln2291Lysfs*10	Frameshift del	LP	–	–	B
*FGD1*	X	c.527dup	p.Leu177Thrfs*40	Frameshift dup	P	P	–	B
*SACS*	13	c.4343del	p.Asn1448Thrfs*15	Frameshift del	LP	–	–	B
*COL4A3*	2	c.4882 T > G	p.Ser1628Ala	Missense	VUS	Conflicting	B	B
*TUBA3D*	2	c.466C > T	p.Arg156Trp	Missense	VUS	–	–	B
*MYH2*	17	c.5045G > A	p.Arg1682His	Missense	VUS	VUS	U	B
*MC1R*	16	c.623_626dup	p.Met210Profs*30	Frameshift dup	VUS	–	–	–
*MKKS*	20	c.1622G > A	p.Cys541Tyr	Missense	VUS	–	U	B
*CNGA3*	2	c.827 A > G	p.Asn276Ser	Missense	P	LP	B	B
*MERTK*	2	c.2302G > A	p.Ala768Thr	Missense	P	LP	D	B
*USH2A*	1	c.6937G > T	p.Gly2313Cys	Missense	P	P	U	B
*IQCB1*	3	c.766 + 1G > A	–	Splice donor	LP	–	–	D
*CFI*	4	c.1305del	p.Asp436Ilefs*49	Frameshift del	P	–	–	B
*USP45*	6	c.1921_1925del	p.Val641Glnfs*20	Frameshift del	LP	–	–	–
*PCK2*	14	c.133C > T	p.Arg45X	Stop-gain	LP	–	–	B
*SLC24A1*	15	c.847G > A	p.Val283Met	Missense	VUS	–	D	B

Abbreviations: P, pathogenic; LP, likely pathogenic; VUS, variant of uncertain significance; B, benign; D, deleterious; U, uncertain. AlphaMissense and SpliceAI columns report in-silico predictions. * Single-allele finding (ABCA4 start-loss; see Table 2, footnote d).

## 4 Discussion

### 4.1 Principal findings

In this consecutive series of 30 previously unresolved Iranian probands with congenital or early-onset ocular disorders, WES established a molecular diagnosis in 70%. The spectrum was dominated by autosomal-recessive IRD genes, and two-thirds of genotypes were homozygous or hemizygous, mirroring the consanguinity in roughly one-third of families. These findings position WES as an effective first-tier test while defining a clear residual gap: one-third of probands remained unsolved, and a further subset were only partially resolved by single-allele findings.

### 4.2 Diagnostic yield in the context of published cohorts

Our 70% yield should be read against a methodologically heterogeneous literature rather than taken at face value. A 2023 meta-analysis of NGS in IRD reported pooled yields of 52–74%, rising to 73.5% for exome-based studies and 65.6% for studies applying ACMG criteria [6]; our estimate sits at the upper end of this range. Within Iran, higher yields of ≈82% [13] and 72.9% in 192 consanguineous families [14] have been reported. Two methodological differences — rather than any biological distinction — most plausibly explain this gap. First, those studies incorporated family segregation and, in the larger cohort, autozygosity mapping and copy-number detection, with copy-number variants alone contributing 5.3% of diagnoses [14]; we performed none of these. Second, yield is strongly modified by ascertainment: it rises with earlier onset and with the number of affected relatives and falls in singleton, proband-only designs, where confirmed exome yields at tertiary centres have been as low as 24–30% [22]. Our cohort's enrichment for infantile-onset disease (73%) and consanguinity (37%) therefore likely raised the yield, whereas the proband-only design and absence of copy-number analysis lowered it — a tension that frames 70% as a credible but conservative estimate.

### 4.3 Variant spectrum and the consanguineous genome

The predominance of homozygous and hemizygous genotypes is the expected genomic signature of consanguinity and, on its own, is unremarkable: in consanguineous IRD cohorts the great majority of pathogenic variants fall within runs of homozygosity [8], [14]. Its value lies not in novelty but in identifying the recurrent genes and alleles relevant to Iranian families — *ABCA4, CRB1, CEP290, RP1* and the BBS-pathway genes dominated our spectrum, mirroring national [10], [12], [23] and international [24], [25] data. The comparatively high frequency of syndromic IRDs, particularly Bardet–Biedl syndrome, is consistent with founder effects and the informativeness of multiplex consanguineous pedigrees [20], [21]. We did not, however, formally analyse runs of homozygosity or founder haplotypes, and therefore cannot claim specific founder effects in this cohort.

### 4.4 Single-allele findings and the limits of exome sequencing

The most clinically consequential pattern was the single heterozygous pathogenic allele in an autosomal-recessive gene, seen in roughly one-third of genotypes. In consanguineous families this is paradoxical, and is most parsimoniously explained not by non-genetic disease but by a second allele that exome sequencing cannot capture — a deep-intronic, regulatory, or copy-number variant. The supporting evidence is substantial: copy-number variation accounts for ≈9% of IRD pathogenicity [26]; genome sequencing of exome-negative cases recovers a further 13–29% of diagnoses, predominantly through structural and deep-intronic variants [27], [28], [29], [30]; and structural variants are systematically under-ascertained by short-read exomes [31], [32]. Reanalysis alone, without new sequencing, adds a further 8–25% [33], [34]. An appraisal must, however, weigh the ceiling of these approaches: the incremental yield of genome sequencing in exome-negative IRD cohorts is often modest and concentrated in a few recurrent deep-intronic or structural alleles rather than reflecting broad genome-wide discovery [28], [29]. Critically, we performed no copy-number, structural-variant, or genome sequencing; our inference that single-allele findings reflect undetected second alleles is therefore a testable hypothesis, not a demonstrated result — the central interpretive caveat of this study.

### 4.5 Caveats in variant–phenotype assignment

Several individual assignments warrant caution and were treated conservatively. The single *ABCA4* start-loss allele is non-diagnostic for autosomal-recessive RP19 in the absence of a second variant; the *GOLGA8M* variant lies in a gene not established in retinal disease, the RP11 locus mapping instead to *PRPF31* on chromosome 19; and several findings — in *KMT2A, SACS, COL4A3, MYH2* and *MC1R* — involve genes whose ocular manifestations are secondary to systemic disease and therefore inform syndromic surveillance rather than a primary retinal diagnosis. Treating such variants as definitive would inflate the apparent yield, which we have avoided.

### 4.6 Clinical and counselling implications

Three counselling messages follow. First, a negative or single-allele exome result does not exclude a recessive diagnosis; families should be counselled accordingly and offered reflex genomic testing. Second, the recurrence of syndromic IRDs mandates systematic evaluation of extra-ocular features at molecular diagnosis. Third, accurate interpretation in this population depends on population-matched allele frequencies; our use of the Iranian GEMIRAN and IRANOM resources alongside gnomAD illustrates the value of national databases for an under-represented population whose variant spectrum is incompletely captured by global references [14], [35].

### 4.7 Strengths and limitations

Strengths include consecutive ascertainment, population-specific allele-frequency resources, dual variant callers, and two-reviewer ACMG classification with transparent reporting of incomplete genotypes. The limitations bear directly on interpretation: a modest, single-centre sample; a proband-only design with limited segregation data; the absence of copy-number, structural-variant, deep-intronic, and genome sequencing; the lack of orthogonal confirmation for every variant; and the descriptive, non-comparative design. Because runs of homozygosity and founder haplotypes were not analysed, population-genetic inferences such as founder effects remain beyond the reach of these data.

### 4.8 Future directions

Priorities for subsequent work follow from these limitations: exome-based and dedicated copy-number/structural-variant calling, deep-intronic and RNA-splicing assays, systematic family segregation, and reflex genome sequencing for unsolved and partially solved cases [28], [29], [36], together with autozygosity and founder-haplotype analyses to exploit the informativeness of consanguineous pedigrees and to expand Iranian allele-frequency resources [8], [14].

## 5 Conclusion

WES provided a molecular diagnosis in approximately 70% of previously unresolved Iranian probands with congenital or early-onset ocular disorders, supporting its use as a first-tier test in this high-consanguinity population. A substantial minority remained partially or fully unresolved, chiefly because of single heterozygous findings in recessive genes that exome sequencing cannot phase or that may reflect undetected copy-number or deep-intronic variants. A structured, iterative strategy — first-tier WES, transparent reporting of incomplete genotypes, periodic reanalysis, and reflex copy-number and genome sequencing — is therefore required to maximise yield and support accurate counselling and precision management.

## CRediT authorship contribution statement

**Ali Asadi:** Writing – review & editing, Writing – original draft, Visualization, Validation, Resources, Methodology, Investigation, Formal analysis, Data curation, Conceptualization, Project administration. **Seyed Ataollah Sadat Shandiz:** Writing – review & editing, Supervision. **Amirhossein Ebrahimi:** Writing – original draft. **Zahra Sadat Hosseini:** Formal analysis, Visualization. **Niyousha Shirsalimi:** Writing – original draft. **Hossein Neamatzadeh:** Methodology. **Ahmad Ebrahimi:** Validation, Methodology, Resources, Supervision, Writing – review & editing.

## Consent for publication

All participants (or their legal guardians) provided written consent for publication of de-identified genetic and clinical data.

## Ethics approval and consent to participate

Approved by the institutional Ethics Committee (IR.IAU.CTB.REC.1403.097) and conducted per the Declaration of Helsinki. Written informed consent was obtained from all participants or their legal guardians.

## Funding

This research received no external funding. DNA extraction, sequencing, and bioinformatic services were provided by Genia Medical Genetics Center and Omics Company, which had no role in study design, interpretation, or the decision to publish.

## Declaration of competing interest

The authors declare no competing interests. Affiliations with Genia Medical Genetics Center and Omics Company are disclosed in the Funding statement.

## Data Availability

The de-identified variant and phenotype dataset is available in the Zenodo repository at https://doi.org/10.5281/zenodo.18436791. Deposited variables include HGVS nomenclature, ACMG classification, ClinVar status, zygosity, and phenotype codes (four data files and a README).

## Appendix A Supplementary data

Suppl. Table S1. Aggregate exome sequencing and quality-control metrics for the 30-proband cohort. Suppl. Table S2. ACMG/AMP 2015 criteria applied to variant classification.
