# Supplementary material for: Diagnostic yield and variant spectrum of whole-exome sequencing in Iranian probands with congenital and early-onset ocular disorders
Source: Mol Genet Metab Rep. 2026 Jul 20;48:101339. doi: 10.1016/j.ymgmr.2026.101339 (PMC13393353; doi:10.1016/j.ymgmr.2026.101339)
Supplement: Supplementary file 1 — Suppl. Table S1. Aggregate exome sequencing and quality-control metrics for the 30-proband cohort. Suppl. Table S2. ACMG/AMP 2015 criteria applied to variant classification. [file mmc1.docx]

**Supplementary Material**

*Whole-Exome Sequencing in Iranian Probands with Congenital and Early-Onset Ocular Disorders*

# Supplementary Table S1. Sequencing and quality-control summary

Aggregate exome quality-control metrics for the 30-proband cohort. Per-sample metrics are deposited with the variant dataset in the Zenodo repository (doi:10.5281/zenodo.18436791).

| **Parameter** | **Value** |
| --- | --- |
| **Capture kit** | Agilent SureSelect Human All Exon V7 |
| **Sequencer / read length** | Illumina NovaSeq 6000; 2 × 150 bp paired-end |
| **Reference genome** | GRCh38/hg38 |
| **Mean on-target depth (cohort)** | ≈99–100× |
| **Target bases covered ≥20×** | ≈95% |
| **Target bases covered ≥10×** | ≈98% |
| **Duplicate rate (typical)** | 8–12% |
| **Read aligner** | BWA-MEM v0.7.17 |
| **Variant callers** | GATK HaplotypeCaller v4.2; DeepVariant v1.4 |
| **Variant filtering** | GATK VQSR |
| **Read pre-processing** | SOAPnuke (adaptor/contaminant/low-quality removal, pre-alignment) |
| **Annotation databases** | gnomAD v4; GEMIRAN; IRANOM; ClinVar; OMIM |
| **In-silico predictors** | CADD; REVEL; SpliceAI; AlphaMissense |
| **Classification framework** | ACMG/AMP 2015 (two independent reviewers) |

# Supplementary Table S2. ACMG/AMP criteria applied to reported variants

Key ACMG/AMP 2015 criteria supporting the classification of each reported variant in Table 2. PVS1 applies to null variants in genes with established loss-of-function mechanism; PM2 reflects rarity/absence in population databases; PM3 applies to recessive variants detected (or expected) in trans; PP3 reflects concordant in-silico prediction; PP1 reflects co-segregation where available. Classifications are conservative where evidence is incomplete.

| **Gene** | **Variant** | **Class** | **Type** | **ACMG criteria** |
| --- | --- | --- | --- | --- |
| **RP1** | c.788-1G>A | LP | Splice acceptor (±1) | PVS1, PM2, PP3 |
| **PAX6** | c.730_731del | LP | Frameshift | PVS1_moderate, PM2 (ClinVar conflicting) |
| **BBS10** | c.947del | LP | Frameshift | PVS1, PM2 |
| **POLA1** | c.3550T>G | VUS | Missense (X-linked) | PM2, PP3 (multisystem phenotype) |
| **OFD1** | c.710del | P | Frameshift (hemizygous) | PVS1, PM2, PP4 |
| **CFAP418** | c.394T>C | VUS | Missense | PM2, PP3 |
| **KCNV2** | c.995_996dup | P | Frameshift | PVS1, PM2, PP4 |
| **ATAD3A** | c.178A>T | LP | Stop-gain | PVS1, PM2 |
| **ABCA4** | c.1A>G | VUS* | Start-loss (single allele) | PVS1_moderate, PM2 (single allele → non-diagnostic) |
| **KMT2A** | c.5993_5996del | P | Frameshift | PVS1, PM2 |
| **GUCY2D** | c.2828dup | LP | Frameshift | PVS1, PM2 |
| **GOLGA8M** | c.1321C>T | VUS | Stop-gain (gene not disease-validated) | PM2 (no established gene–disease relationship) |
| **CRYAA** | c.124T>C | VUS | Missense | PM2, PP3 |
| **CRB1** | c.3158G>A | LP | Missense | PM2, PP3, PM1 |
| **ADGRV1** | c.14197C>T | LP | Stop-gain | PVS1, PM2 (digenic context) |
| **PDZD7** | c.1079G>A | VUS | Missense | PM2, PP3 (digenic context) |
| **BBS12** | c.265_266del | P | Frameshift | PVS1, PM2, PP4 |
| **CEP290** | c.6870del | LP | Frameshift | PVS1, PM2 |
| **FGD1** | c.527dup | P | Frameshift (hemizygous) | PVS1, PM2, PP4 |
| **SACS** | c.4343del | LP | Frameshift | PVS1, PM2 |
| **COL4A3** | c.4882T>G | VUS | Missense | PM2 (ClinVar conflicting) |
| **TUBA3D** | c.466C>T | VUS | Missense | PM2, PP3 (limited evidence) |
| **MKKS** | c.1622G>A | VUS | Missense | PM2, PP3 |
| **TYR** | c.139G>T / c.1430G>A | LP/LP | Missense + nonsense (compound het) | PVS1 (nonsense), PM3, PM2, PP3 |
| **MYH2** | c.5045G>A | VUS | Missense | PM2, PP3 |
| **MC1R** | c.623_626dup | VUS | Frameshift (incidental) | PM2 (non-ocular gene) |

** ABCA4 c.1A>G is a single-allele finding in an autosomal-recessive gene and is therefore non-diagnostic in isolation; a second allele was not detected on exome data.*
